# Comprehensive Genetic Results for Primary Immunodeficiency Disorders in a Highly Consanguineous Population

**DOI:** 10.3389/fimmu.2018.03146

**Published:** 2019-01-15

**Authors:** Waleed Al-Herz, Janet Chou, Ottavia Maria Delmonte, Michel J. Massaad, Wayne Bainter, Riccardo Castagnoli, Christoph Klein, Yenan T. Bryceson, Raif S. Geha, Luigi D. Notarangelo

**Affiliations:** ^1^Department of Pediatrics, Faculty of Medicine, Kuwait University, Kuwait City, Kuwait; ^2^Allergy and Clinical Immunology Unit, Pediatric Department, Al-Sabah Hospital, Kuwait City, Kuwait; ^3^Division of Immunology, Department of Pediatrics, Children's Hospital, Harvard Medical School, Boston, MA, United States; ^4^Laboratory of Clinical Immunology and Microbiology, Division of Intramural Research, National Institute of Allergy and Infectious Diseases, National Institutes of Health, Bethesda, MD, United States; ^5^Department of Experimental Pathology, Immunology, and Microbiology, Pediatrics and Adolescent Medicine, Faculty of Medicine, American University of Beirut, Beirut, Lebanon; ^6^Department of Pediatrics, University of Pavia, Foundation IRCCS Policlinico San Matteo, Pavia, Italy; ^7^Department of Pediatrics, Dr. von Hauner Children's Hospital, University Hospital, LMU, Munich, Germany; ^8^Department of Medicine, Centre for Hematology and Regenerative Medicine, Karolinska Institutet, Karolinska University Hospital Huddinge, Stockholm, Sweden

**Keywords:** primary immunodeficiencies, genetic, consanguinity, autosomal recessive, mutation

## Abstract

**Objective:** To present the genetic causes of patients with primary immune deficiencies (PIDs) in Kuwait between 2004 and 2017.

**Methods:** The data was obtained from the Kuwait National Primary Immunodeficiency Disorders Registry. Genomic DNA from patients with clinical and immunological features of PID was sequenced using Sanger sequencing (SS), next generation sequencing (NGS) of targeted genes, whole exome sequencing (WES), and/or whole genome sequencing (WGS). Functional assays were utilized to assess the biologic effect of identified variants. Fluorescence *in situ* hybridization (FISH) for 22q11.2 deletion and genomic hybridizations arrays were performed when thymic defects were suspected.

**Results:** A total of 264 patients were registered during the study period with predominance of patients with immunodeficiencies affecting cellular and humoral immunity (35.2%), followed by combined immunodeficiencies with associated syndromic features (24%). Parental consanguinity and family history suggestive of PID were reported in 213 (81%) and 145 patients (55%), respectively. Genetic testing of 206 patients resulted in a diagnostic yield of 70%. Mutations were identified in 46 different genes and more than 90% of the reported genetic defects were transmitted by in an autosomal recessive pattern. The majority of the mutations were missense mutations (57%) followed by deletions and frame shift mutations. Five novel disease-causing genes were discovered.

**Conclusions:** Genetic testing should be an integral part in the management of primary immunodeficiency patients. This will help the delivery of precision medicine and facilitate proper genetic counseling. Studying inbred populations using sophisticated diagnostic methods can allow better understanding of the genetics of primary immunodeficiency disorders.

## Introduction

Primary immunodeficiency diseases (PIDs) comprise a heterogeneous group of monogenic inborn errors of immunity with a wide spectrum of clinical manifestations. Due to international collaborative efforts and the availability of advanced functional and genetic testing, our knowledge of the underlying causes of PIDs has tremendously increased in the last decades (1). However, the prevalence of PIDs remains underestimated due to lack of awareness among general practitioners (2). Previous reports have shown great ethnic and geographic variability in the prevalence and distribution of PIDs (3–8), probably related to the higher frequency of consanguineous marriages and to the presence of founder effects in certain populations.

The evaluation of patients suspected to have PIDs starts with reviewing the clinical presentation, followed by phenotypic and functional immunologic studies. Because of the vast clinical and immunologic heterogeneity of patients' mutations in known causative genes (9), and the large number of potentially causative mutations in novel genes, genetic testing has assumed increasing importance in the diagnosis and management of PIDs (10). Early molecular diagnosis provides the opportunity for timely treatment such as antibody replacement and hematopoietic stem cell transplantation, which may help prevent disease-related complications and death. Genetic diagnoses also enable the use of targeted therapies such as immune suppression, treatment with monoclonal antibodies or specific small molecule inhibitors, or gene therapy which may also significantly improve the patients' outcome (11, 12). Furthermore, genetic diagnosis facilitates genetic counseling, prenatal, and preimplantation genetic diagnosis, and pre-marital carrier testing. Finally, it provides important information about phenotype-genotype correlation, with important prognostic and therapeutic implications (13, 14).

In this report, we present the results of genetic studies performed in a large cohort of PIDs patients from Kuwait between 2004 and 2017.

## Materials and Methods

### Patients Data

The data was obtained from the Kuwait National Primary Immunodeficiency Disorders Registry (KNPIDR) which was approved by The Research and Ethics Committee of the Ministry of Health in Kuwait in accordance with the Declaration of Helsinki. An informed written consent was obtained from patients and/or families for whom the genetic testing was done for research purposes, but such consent was not taken for tests done for clinical purposes. PIDs were classified according to the International Union of Immunological Societies, Primary Immunodeficiency Diseases Committee report on Inborn Errors of Immunity (2017) (1). A consanguineous marriage was defined as one in which two partners had at least one ancestor in common, with the ancestor being no more distant than a great grandparent (15).

### Genetic Testing

Genomic DNA was extracted from whole blood. Where patients presented with classical clinical and immunological features or in order to confirm next generation sequencing (NGS) findings, targeted Sanger sequencing (SS) of gene(s) of interest was performed on genomic DNA according to standard protocols (IRB-approved protocol 16-I-N139 for patients analyzed at the NIH). Fluorescence *in situ* hybridization (FISH) for 22q11.2 deletion and genomic hybridization array were performed when thymic defects were suspected (16). In multiple patients, NGS was used as first line strategy for genetic testing. NGS was performed using the PID v2 panel and Ion Torrent S5 sequencer (ThermoFisher, Waltham, MA), with an average coverage of 253x. Coverage analysis and variant calling was performed using Ion Reporter software (ThermoFisher) (17). For whole exome sequencing (WES), exome capture was performed using the SureSelect Human All Exon v4+UTR kit (Agilent Technologies). A HiSeq 2000 system (Illumina) was used to generate 100 base-pair paired-end reads, with an average on-target coverage of 80x. In some individuals, whole exome sequencing was done on Illumina Next Seq platforms, with coverage of 100x. The presence of large deletions was detected by copy number variation analysis of WES data (18). Reads were aligned to the GRCh37 reference assembly human genome using BWA Single nucleotide variants and indels were detected with GATK using standard hard filtering parameters. Variants with a read coverage <2x and a Phred-scaled SNP quality of ≤20 were eliminated. Whole genome sequencing (WGS), read mapping, local *de novo* assembly, and variant calling and annotation were performed by Complete Genomics, Inc. We have divided the mutations according to the type and each mutation was considered individually in the final mutation enumeration.

## Results

### Patients Distribution, Parental Consanguinity, and Familial History

**Figure 1:**
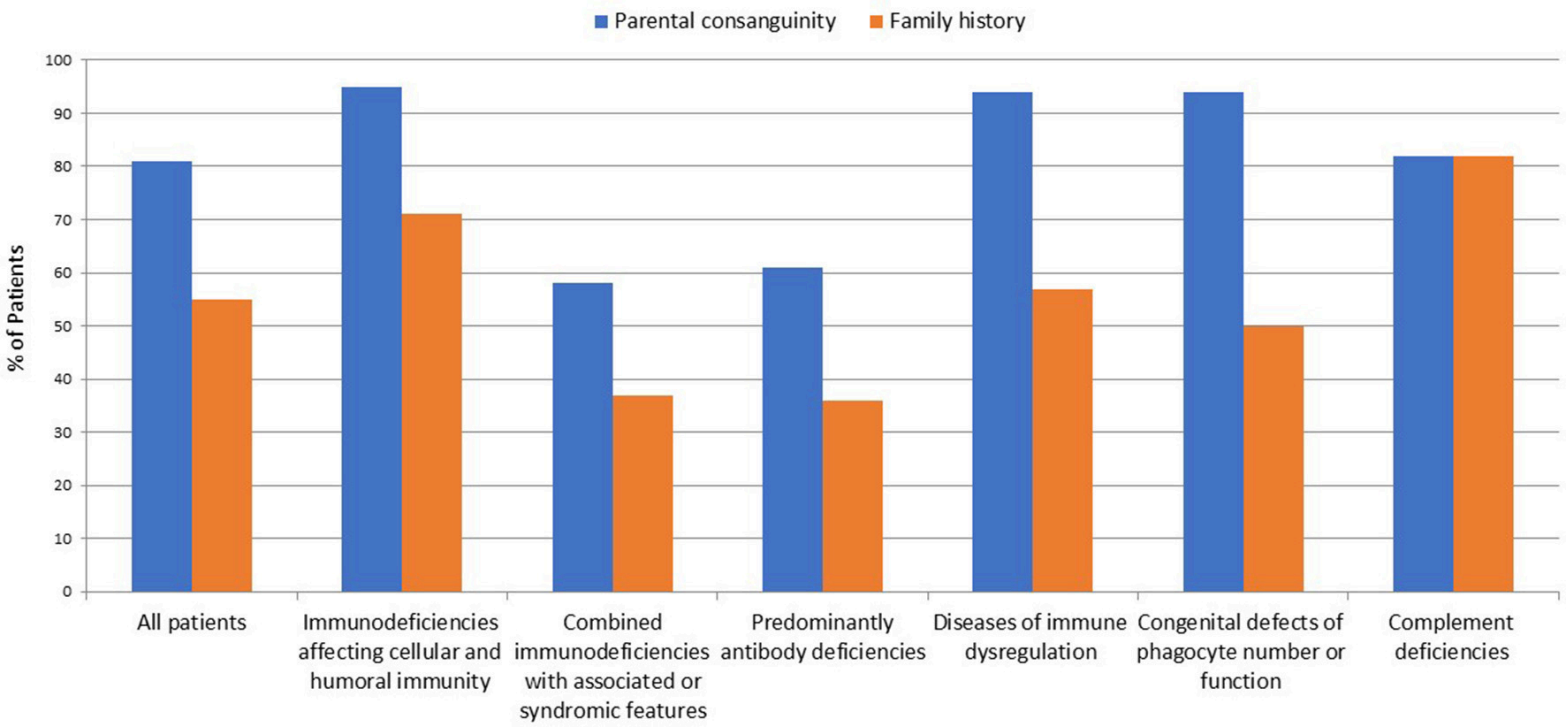
Frequency of parental consanguinity and family history among 264 patients registered in KNPIDR.

A total of 264 patients were registered in KNPIDR from January 2004 to December 2017 and distributed as follows: immunodeficiencies affecting cellular and humoral immunity, 93 patients (35.2%); combined immunodeficiencies (CID) with associated syndromic features, 64 patients (24.3%); predominantly antibody deficiencies, 33 patients (12.5%); diseases of immune dysregulation, 46 patients (17.5%); congenital defects of phagocyte number or function, 16 patients (6.1%); autoinflammatory disorders, 1 patient (0.3%%); and, complement deficiencies, 11 patients (4.1%). The diagnosis was established in four patients based on lack of protein expression as assessed by flow cytometry (three patients with MHC II deficiency and one patient with LAD-1 deficiency). Parental consanguinity and family history suggestive of PID were reported in 213 (81%) and 145 patients (55%), respectively (Figure 1). The presence of family history of PID prompted pre-symptomatic immunologic and/or genetic testing in 31 patients (12%). Thirty patients were diagnosed with 22q11.2 deletions by FISH study.

### Genetic Results

**Figure 2:**
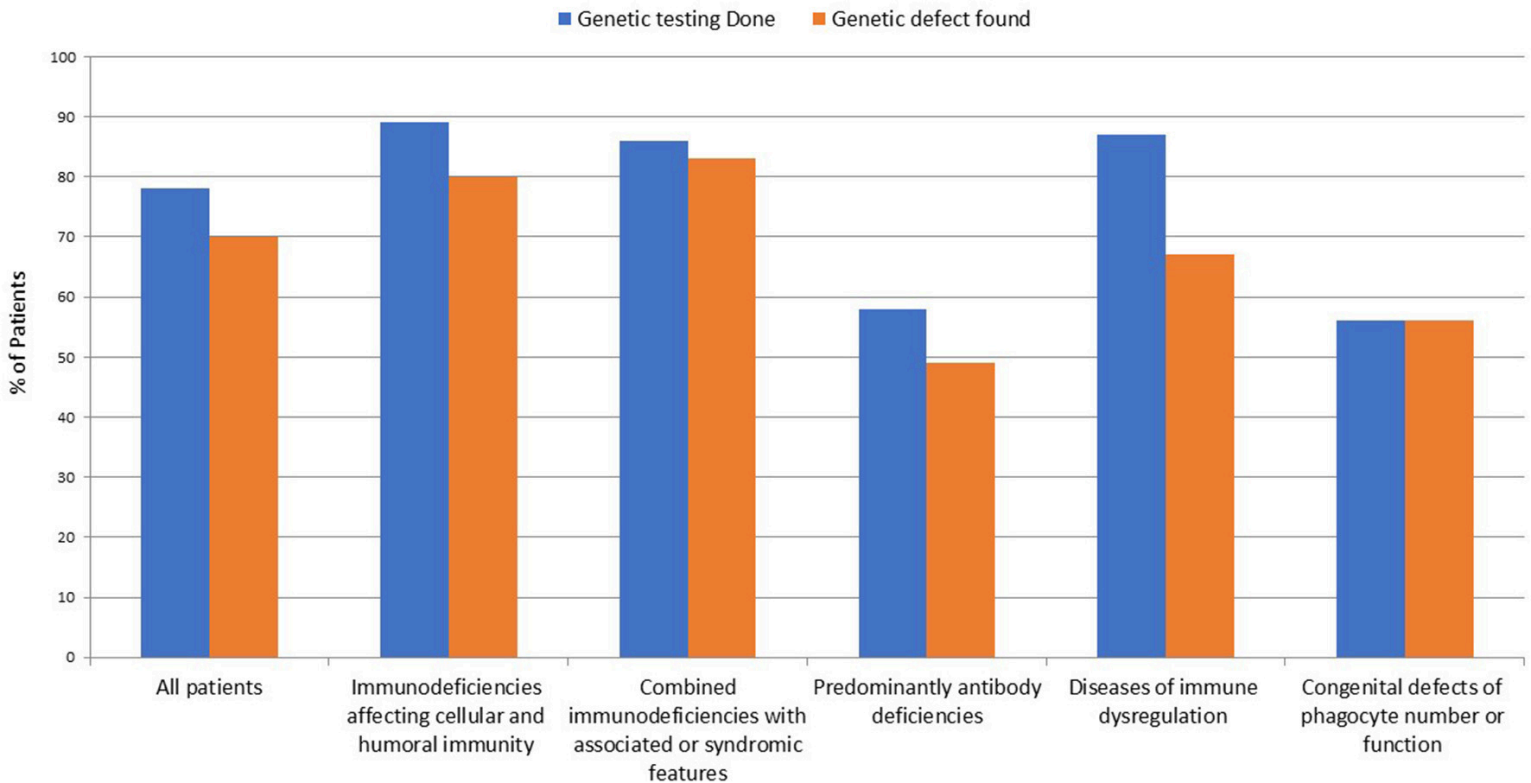
Frequency of genetic testing among 264 patients registered in KNPIDR.

**Figure 3:**
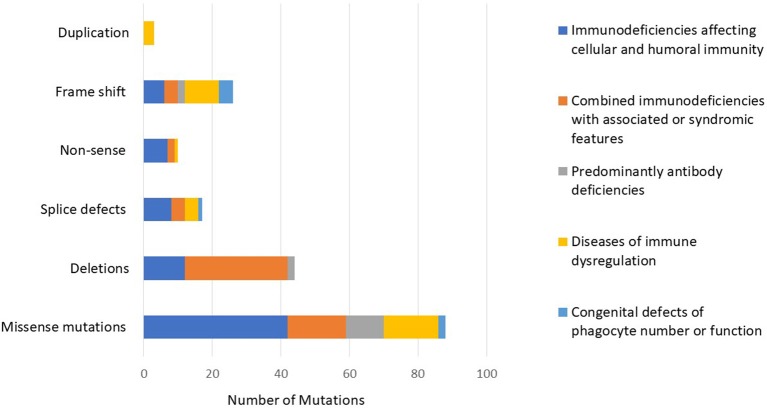
The distribution of mutations according to the PID category showing a predominance of missense mutations followed by deletions and frame shift mutations.

The number of patients who underwent genetic testing was 206 (78%) with an overall diagnostic yield of 70% (184 patients) (Figure 2 and Table 1). Genetic testing was not attempted for 58 patients due to various reasons like unavailability of such testing at diagnosis or the patients' death. The numbers of patients diagnosed by FISH and Sanger sequencing were 30 and 99, respectively, while 44 and 11 patients were diagnosed by whole exome and whole genome sequencing, respectively. None of the patients with autoinflammatory disorders or complement deficiencies underwent genetic testing. The majority of the mutations were missense mutations (57%) followed by deletions and frame shift mutations (Figure 3). Five patients are known to have specific genetic defects, but the exact mutations are not available at the time of writing this manuscript (3 patients with CSF2RB, 1 patient with WAS, and 1 patient with BTK mutations).

**Table 1:** Mode of inheritance and molecular studies in the 184 patients with known genetic defect.

**Gene**	**Mode of inheritance**	**Number of patients**	**Molecular defects**		**Genetic analysis performed to first detect mutation**
			**cDNA**	**Protein**	
**IMMUNODEFICIENCIES AFFECTING CELLULAR AND HUMORAL IMMUNITY (*****N*** **=** **75)**					
RAG1	AR	7	c.1361T>A	L454Q	6 SS−1 WES
		1	–	R404Q	WGS
		1	–	R394W	SS
RAG2	AR	6	–	G35A	4 SS−2 WES
DCLRE1C	AR	3	–	G135R	SS
		2	Ex 1-9 del/Ex 1-3 del	–	SS
		1	–	K157KfsX13	SS
		1		G6E	WES
JAK3	AR	2	c.1019C>A	S340X	WES
		2	–	A573P	1 WES– 1 SS
		1	c.1744C>T	R582W	SS
AK2	AR	5	c.524G>A	R175Q	SS
CD3D	AR	3	c.56-3T>G	–	WES
ADA	AR	2	c.428dupA	–	SS
DOCK8	AR	6	Ex 1-5 del	–	SS
		1	c.4070C>A	S1357X	SS
		1	Ex 2-12 del	–	SS
		1	Ex 1-23 del	–	SS
		1	Ex 1-2 del	–	SS
DOCK2	AR	2	c.1868G>A	W623X	WES
		1	–	Y1242YfsX33	WES
RFXANK	AR	6	insTCAC.IVS4+1	–	SS
		4	c.362A>T	D121V	SS
		2	c.564G>A	W188X	SS
ZAP70	AR	1	c.1606G>A	G536S	SS
TFRC	AR	8	–	Y20H	WGS
IKBKB	AR	1	c.736A>G	S246G	WES
ICOS	AR	2	c.90delG	M30fsX26	WES
GM2A	AR	1	c.Chr5q33.1 del	–	CMA
**COMBINED IMMUNODEFICIENCIES WITH ASSOCIATED OR SYNDROMIC FEATURES (*****N*** **=** **54)**					
WAS	XL	1	c.400G>A	A134T	SS
		1	c.91G>A	E31K	SS
		1	–	–	SS
ATM	AR	3	c.381delA/ IVS 44+1 G>A c.2932T>C (Het)	– S978P	SS
		2	c.7082T>C	L2360P	SS
		1	c.748C>T	R250X	SS
22q11.2DS	AD	30	22q11.2 del	–	FISH–CMA
STAT3	AD LOF	2	c. 1144C>T	R382W	SS
		1	c.1910T>C	V637A	SS
		1	c. 1868G>T	W623L	SS
STAT5B	AR	2	c.1643-1delG	–	SS
DNMT3B	AR	1	c.Chr20:31390243G>A	–	WES
ZBTB24	AR	1	c.1492C>A	–	WES
RMRP	AR	1	c.27G>A	–	SS
TTC7A	AR	1	c.1919+1G>A	–	WES
HOIP (RNF31)	AR	1	c.215T>C	L72P	WES
SP110	AR	2	c.617C>T (homozygous) c.642delC (homozygous)	A206V S215fs	SS
MYSM1	AR	2	c.1168G>T	E390X	WES
**PREDOMINANTLY ANTIBODY DEFICIENCIES (*****N*** **=** **15)**					
BTK	XL	1	c.82C>T	R28C	SS
		1	c.982C>T	Q328X	SS
		1	Ex 4–5 del	–	WES
		1	–	–	SS
AICDA	AR	7	c.254G>A	S85N	SS
		2	c.169G>A	V57M	WES
NFKB2	AD	2	c.2596_2597delAG	S866fs	WES
**DISEASES OF IMMUNE DYSREGULATION (n=31)**					
LYST	AR	2	c.1902dupA	A635SfsX4	1 WES−1 SS
		1	IVS19 c.5784+5 G>A	–	SS
		1	c.2311C>T	Q771X	WES
TNFRSF6	AR	2	c.1110 del 290 ins TTGT	L290fs	SS
FASLG	AR	2	c.829G>A	G277S	SS
AIRE	AR	2	–	D70AfsX148	SS
		1	c.274C>T	R92W	SS
PRF1 (FHL2)	AR	2	c.133G>A	G45R	WES
		2	c.1081A>T	R361W	WES
STX11 (FHL4)	AR	2	c.290delG	G97AfsX11	WES
STXBP2 (FHL5)	AR	2	c.1213C>T/c.1247-1G>C	R405W/–	WES
		1	c.1463C>T/c.37+2T>C	P488L/–	WES
		1	c.1430C>T	P477L	WES
XIAP	XL	1	c.146G>A	R49Q	WES
IL10	AR	4	c.458G>A	G153D	SS
RAB27A	AR	2	c.514_518delCAAGC	Q172NfsX2	WES
LRBA	AR	3	Ex 49–53 dup	–	WGS
**CONGENITAL DEFECTS OF PHAGOCYTE NUMBER OR FUNCTION (*****N*** **=** **9)**					
CYBA	AR	3	c.295_301 delGTGCCCG	V99fsX89	SS
NCF1	AR	1	c.73_74delGT	Y26HfsX25	SS
NCF2	AR	1	c.1000+1G>A	P333X353	WES
JAGN1	AR	1	c.51T>G/c.476A>G	F17L/H159R	WES
CSF2RB	AR	3	–	–	SS

*RAG1, Recombination Activating 1; RAG2, Recombination Activating 2; DCLRE1C, DNA Cross-Link Repair 1C; JAK3, Janus Kinase 3; AK2, Adenylate Kinase 2; CD3D, CD3d Molecule; ADA, Adenosine Deaminase; DOCK8, Dedicator Of Cytokinesis 8; DOCK2, Dedicator Of Cytokinesis 2; RFXANK, Regulatory Factor X Associated Ankyrin Containing Protein; ZAP70, Zeta Chain Of T Cell Receptor Associated Protein Kinase 70; TFRC, Transferrin Receptor; IKBKB, Inhibitor Of Nuclear Factor Kappa B Kinase Subunit Beta; ICOS, Inducible T Cell Costimulator; GM2A, GM2 Ganglioside Activator; WAS, Wiskott-Aldrich Syndrome; ATM, ATM Serine/Threonine Kinase; 22q11.2DS, DiGeorge syndrome; STAT3, Signal Transducer And Activator Of Transcription 3; STAT5B, Signal Transducer And Activator Of Transcription 5B; DNMT3B, DNA Methyltransferase 3 Beta; ZBTB24, Zinc Finger And BTB Domain Containing 24; RMRP, RNA Component Of Mitochondrial RNA Processing Endoribonuclease; TTC7A, Tetratricopeptide Repeat Domain 7A; HOIP (RNF31), HOIL-1-Interacting Protein (Ring Finger Protein 31); SP110, SP110 Nuclear Body Protein; MYSM1, Myb Like, SWIRM And MPN Domains 1; BTK, Bruton Tyrosine Kinase; AICDA, Activation Induced Cytidine Deaminase; NFKB2, Nuclear Factor Kappa B Subunit 2; LYST, Lysosomal Trafficking Regulator; TNFRSF6, Fas (TNFRSF6)-Associated Via Death Domain; FASLG, Fas Ligand; AIRE, Autoimmune Regulator; PRF1, Perforin 1; STX11, Syntaxin 11; STXBP2, Syntaxin Binding Protein 2; XIAP, X-Linked Inhibitor Of Apoptosis; IL10, Interleukin 10; RAB27A, RAB27A Member RAS Oncogene Family; LRBA, LPS Responsive Beige-Like Anchor Protein; CYBA, Cytochrome B-245 Alpha Chain; NCF1, Neutrophil Cytosolic Factor 1; NCF2, Neutrophil Cytosolic Factor 2; JAGN1, Jagunal Homolog 1; CSF2RB, Colony Stimulating Factor 2 Receptor Beta Common Subunit*.

Genetic testing was performed in 83 out of 93 patients with immunodeficiencies affecting cellular and humoral immunity. The disease-causing mutation was identified in 75 patients (90%). Among those, 37 (49%) carried defects in genes that cause severe combined immune deficiency (SCID) (1). Causes of SCID included mutations in *RAG1* (*n* = 9), *RAG2* (*n* = 6), *DCLRE1C* (*n* = 7), *JAK3* (*n* = 5), *AK2* (*n* = 5), *CD3D* (*n* = 3), *ADA* (*n* = 2). Only two of these 37 patients had compound heterozygous variants, while 35 had homozygous variants.

The remaining 38 subjects with a definitive genetic diagnosis in this group had defects in genes that results in other forms of combined immunodeficiencies (CID), less profound than SCID. The following gene defects were identified: *DOCK8* (*n* = 10), *DOCK2* (*n* = 3), *TFRC* deficiency (*n* = 8), *ICOS* deficiency (*n* = 2), *RFXANK* resulting in MHC class II deficiency (*n* = 12 mutations), *ZAP-70* (*n* = 1), *IKBKB* (*n* = 1). No cases of hyper-IgM syndrome (HIGM) due to X-linked CD40 ligand (CD40L) deficiency were reported. A patient with clinical features of Omenn syndrome (OS) who did not harbor defects in genes known to be associated with OS, had a homozygous 691 kb deletion at 5q33.1 encompassing the *GM2A* gene associated with GM2-gangliosidosis, which was detected by chromosomal microarray. Interestingly, large deletions were reported in all DOCK8 deficient patients and two patients with Artemis deficiency. All the diseases reported in this category have autosomal recessive (AR) pattern of transmission.

Among the 64 individuals who were diagnosed with combined immunodeficiencies (CID) with associated syndromic features, a molecular defect was identified in 54 patients (84%). The diagnosis of a DNA repair defect syndrome was made in 14 out of 64 patients (21.8%). Ten of them were diagnosed with ataxia telangiectasia, of whom six were confirmed to have *ATM* mutations while no genetic testing was attempted for the other four patients. Four patients had immunodeficiency with centromeric instability and facial anomalies (ICF) due to a mutation in either *DNMT3B* or *ZBTB24*, while two patients have no identifiable mutations in ICF-causing genes despite satisfying clinical, immunologic, and cytogenetic diagnostic criteria. No Nijmegen breakage or Bloom syndrome patients were identified. Eight out of 64 (12.5%) patients in this group carried the clinical diagnosis of hyper IgE syndrome (HIES). In four of them, the molecular diagnosis of *STAT3* loss-of-function was obtained. No patients with *TYK2* or *PGM3* deficiency were identified. Three patients were diagnosed with Wiskott-Aldrich syndrome (WAS) on the basis of genetic testing and clinical phenotype, while 30 patients were diagnosed with DiGeorge syndrome and carried the typical 22q11.21 microdeletions. Nine patients were categorized as having other syndromic immunodeficiencies, in particular: hepatic veno-occlusive disease with immunodeficiency (*n* = 2 with *SP110* mutations), *STAT5B* deficiency (*n* = 2), *MYSM1* deficiency (*n* = 2) cartilage hair hypoplasia (*n* = 1 with *RMRP* mutation), *TTC7A* deficiency (*n* = 1), and HOIP deficiency (*n* = 1 with *RNF31* mutation). Aside from the patients with DiGeorge syndrome, only four patients of the remaining 24 (16.6%) in this category had an autosomal dominant (AD) disease while three (12.5%) had X-linked (XL) disorders. The remaining 17 subjects (70.8%) had diseases due to biallelic mutations.

Thirty-three patients were diagnosed with predominantly antibody deficiency. Seven of these had transient hypogammaglobulinemia of infancy, 5 had IgA deficiency, and four were diagnosed with common variable immunodeficiency. Among the 19 patients in whom genetic testing was performed, a disease-causing mutation was identified in 16 subjects (84.2%). The following genes were found to carry pathogenic mutations: *BTK* (*n* = 4), *AICDA* (*n* = 9), *NFKB2* (*n* = 2). One patient had μ heavy-chain deficiency likely due to a large deletion as shown by lack of amplification of the whole gene. Also in this group of patients, AR inheritance was predominant (*n* = 10 patients), whereas gene defects with AD and XL inheritance were identified in two and four patients, respectively.

Diseases of immune dysregulation were identified on the basis of clinical and immunologic phenotype in 46 patients. Genetic studies were performed in 40 patients. A disease-causing mutation was found in 31 of the 40 studied (67%). Four patients were diagnosed with Chediak-Higashi syndrome. In three of these, an exonic *LYST* mutation was identified while one subject carried a donor splice site mutation. Four patients had ALPS due to *FAS* (*n* = 2) *or FASL* (*n* = 2) biallelic mutations. Early-onset IBD due to *IL10* deficiency was found in four siblings. Familial hemophagocytic lymphohistiocytosis (FHL) with or without hypopigmentation was identified in a relatively high number of patients in this category (24/46). In 13 out of the 24 (52%) a genetic diagnosis was achieved: *PRF1 n* = 4 (FHL2), STX*11 n* = 2 (FHL4), and *STXBP2 n* = 4 (FHL5), *RAB27A n* = 2 (Griscelli syndrome type 2), *BIRC4 n* = 1 (XIAP). Three individuals were diagnosed with autoimmune polyendocrinopathy syndrome-1 (APS-1) and were confirmed to harbor *AIRE* gene mutations. Only 1 case with XL inheritance and 2 cases with AD disease belonged to this group.

Sixteen individuals in the registry were affected by congenital defects of phagocyte number, function or both, and genetic testing successfully identified a causative mutation in nine of them. Among the 6 patients with a diagnosis of CGD based on positive DHR testing, 5 received genetic testing. Of these, 3 had mutations in *CYBA*, 1 in *NCF1*, and 1 in *NCF2*. Of note, no CGD patients with the XL form of the disease were identified. Three patients with pulmonary alveolar proteinosis were found with mutations in the GM-CSF receptor. One patient with severe congenital neutropenia carried compound heterozygous mutation in the *JAGN1* gene. One patient with cyclic neutropenia, 1 with leukocyte adhesion deficiency type 1 (LAD1) and 3 subjects with glycogen storage disorder type 1 b that had early onset neutropenia, were diagnosed by immunologic/metabolic studies without further genetic evaluation. All patients were affected by AR disorders.

One case of Blau syndrome was identified in the category of autoinflammatory disorder based on clinical findings, but genetic testing was not performed. Finally, 11 patients were identified with complement deficiencies, with the most common being C4 deficiency (*n* = 5) followed by hereditary angioedema (*n* = 2) and four patients had impaired complement function, not well charactrerized. None of the subject belonging to this group received genetic testing.

### Identification of Novel PID-causing Genes

Several patients in our cohort presented with unique clinical and immunologic phenotype (Table 2) and were found to harbor mutations in novel PID-causing genes using either WGS or WES.

**Table 2:** Clinical and immunologic phenotype of patients with novel PID-causing genes.

**Genetic defect OMIM**	**Inheritance**	**T cells**	**B cells**	**Immunoglobulins**	**Associated features**
*TFRC* 616740	AR	Normal number Poor proliferation	Normal number low memory B cells	Low	Recurrent infections, neutropenia, thrombocytopenia, increased TfR1 expression on cell surface
*DOCK2* 603122	AR	Low	Normal	Low	Defective NK degranulation, poor interferon responses in hematopoietic and non-hematopoietic cells
*MYSM1* 612176	AR	Low reduced naïve T cells	Immature B cells	Low	Short stature, recurrent infections, congenital bone marrow failure, myelodysplasia, skeletal anomalies, cataracts, developmental delay
*RNF31* 612487	AR	Low but normal proliferation	Normal, decreased memory B cells	Low	Bacterial and viral infections, autoinflammation, amylopectinosis, lymphangiectasia., impaired NF-κB activation
*NEIL3* 608934	AR	Normal	Normal	Low	Increased lymphocyte apoptosis, autoantibodies, autoimmunity

First, using Whole Genome Sequencing we were able to diagnose multiple members of the same family with *TFRC* deficiency (19). This newly described disorder is due to *TFRC* gene mutations that lead to impaired function of the transferrin receptor 1 protein (TfR1). The patients were cured with hematopoietic stem cell transplantation.

Second, in an international collaborative effort, we discovered that biallelic *DOCK2* (OMIM 603122) mutations cause a novel T^−^B^+^ CID (20).

Third, using genome-wide homozygosity mapping and WES, we demonstrated *MYSM1* (OMIM 612176) mutations in two siblings who presented with congenital bone marrow failure and myelodysplasia (21). Both patients were cured with hematopoietic stem cell transplantation.

Fourth, we studied a patient who was born to related parents and presented with autoinflammation and combined immunodeficiency. She was found to have a missense mutation in *RNF31* causing impaired HOIP expression and destabilization of the LUBAC complex and (22).

Finally, we identified a homozygous missense mutation in the gene encoding the base excision repair enzyme Nei endonuclease VIII-like 3 (*NEIL3*) that abolished enzymatic activity in three siblings from a consanguineous family (23).

**Figure 4:**
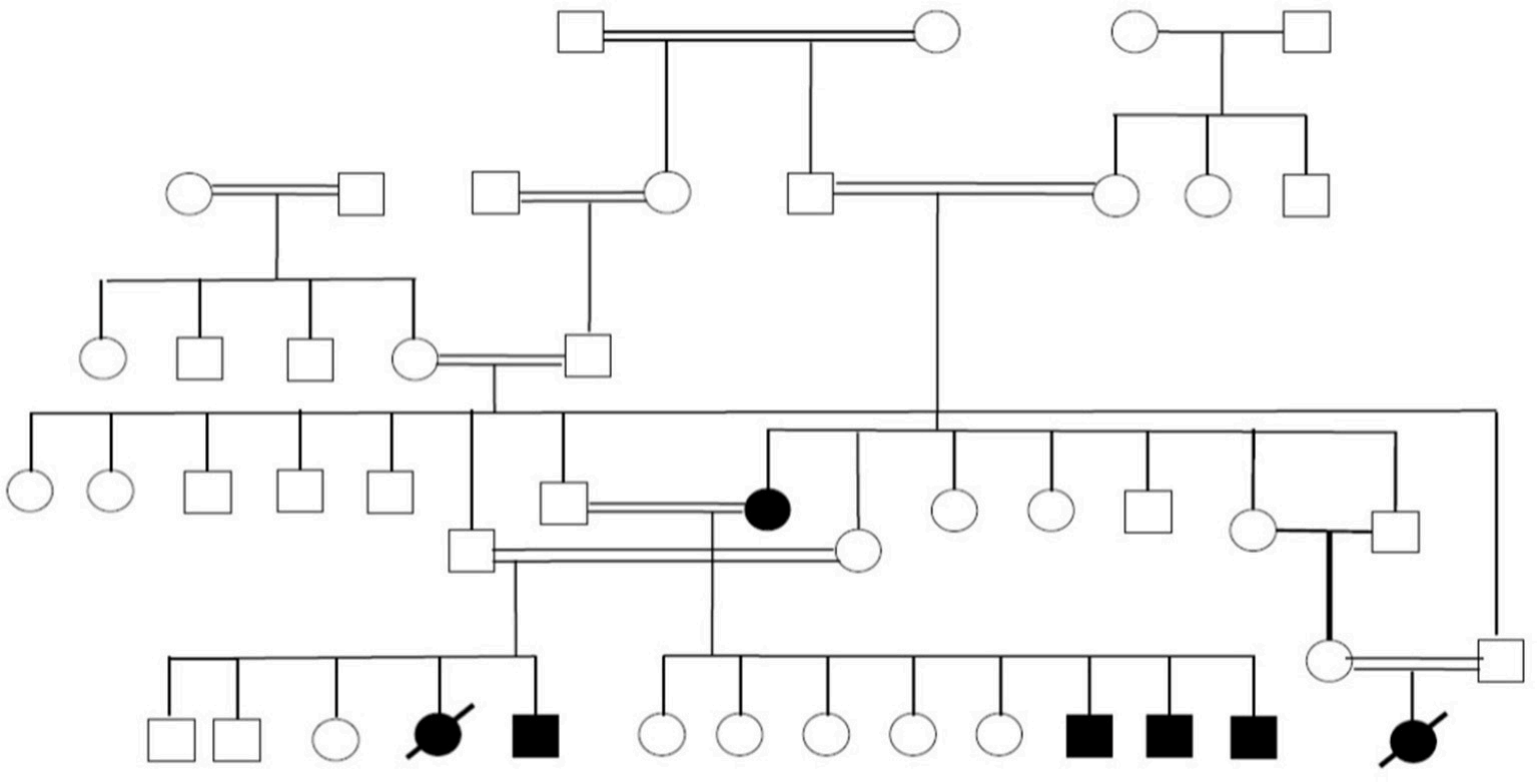
Pedigree showing multiple members of a large family affected with autoimmunity and lymphoproliferation under work-up to identify the underlying genetic cause.

A few other families are currently under work-up to identify potential novel PID-causing genes. An example is our studies in the past few years of a large family with seven individuals affected by a syndrome characterized by autoimmunity and lymphoproliferation (Figure 4). One of the affected subjects has common variable immunodeficiency and another one has selective IgA deficiency.

## Discussion

In this manuscript we present genetic data collected from 184 out of 264 patients who were registered in KNPIDR from January 2004 to December 2017. To the best of our knowledge, this is one of the largest molecular studies of PID patients from a highly consanguineous population along with other studies from the region (24–27). The most represented groups in this series include combined T and B cell immunodeficiencies (157/264) (60%) and disorders of immune dysregulation (46/264) (17.4%). This figure contrasts with data from European series, which showed predominance of antibody deficiencies (7, 8). Indeed, only 12.5% of the patients in the KNPIDR had predominantly antibody deficiencies. Similar observations have been reported in other series from the Middle East and North Africa (MENA) (28, 29).

A prominent finding in our cohort is the relatively high frequency of parental consanguinity (81%) and family history of PIDs (55%), including early deaths, similar clinical phenotype, and/or previously identified PIDs. Another important finding is the high percentage of AR diseases accounting for more than 90% of all PIDs where a genetic diagnosis was achieved, while XL and AD pattern of transmission were detected in only eight patients and six patients (excluding Di George syndrome), respectively. Similarly, molecular studies confirmed that the AR mode of inheritance is the most common in Tunisian patients accounting for 73% of all PIDs entities investigated (25). This is due to the common practice of consanguineous marriages which exceeds 50% in many countries of the MENA region. In Kuwait, parental consanguinity in the general population was 54% (30), while in the KNPIDR it reaches 81% and is particularly high in patients with immunodeficiencies affecting cellular and humoral immunity (94%), disorders of immune dysregulations (94%), and disorders of phagocytes (95%).

Consistent with the high frequency of parental consanguinity, compound heterozygous mutations were found in only 9 of the 140 patients with AR forms of PID. Two of the nine patients were products of consanguineous marriages while seven patients had no history of parental consanguinity. Furthermore, for PIDs with more than one known mode of transmission, the AR mode of inheritance was the most represented in our setting as compared to other series and registries from non-consanguineous populations. While XL SCID is the most represented form of SCID in the United States (31), in this cohort no patients with *IL2RG* deficiency were detected. In line with previous data on CGD from consanguineous populations (25, 32), all genetically diagnosed CGD patients in this report carried mutations in autosomal genes. By contrast, the XL form caused by *CYBB* gene mutation is the most common CGD form in Western countries affecting approximately two-thirds of all patients (33). Similarly, all patients with HIGM in our cohort had an AR form of the disease due to mutations in *AICDA* gene. By contrast, the XL CD40L deficiency has been documented as the most frequent type of HIGM in Asian, and North and Latin American patients (34–36). Thus, AR and XL forms for these diseases should be equally suspected in males originating from consanguineous regions. Furthermore, among 13 cases of genetically confirmed FHL, we observed only one case of XL XIAP deficiency while the remaining 12 patients had AR disorders.

The overall genetic diagnostic yield in our study was 70%. This is slightly lower than a yield of 81% in a study done in Iran, but higher than the yield in studies done in New Zealand (23%) and China (40%) (26, 37, 38). This can be due to differences in the technology used for genetic sequencing studies, the study population, and the number of genes studied. For example, the Iranian study focused on patients with CID only while the study from New Zealand included a wide spectrum of diseases like aHUS/C3 glomerulopathy, type III HAE, suspected HLH, aHUS or periodic fever syndromes. Moreover, Sanger sequencing was the only technique used to search for genetic defects in the study from China, which may explain the lower rate of gene defects identified, especially among patients with atypical clinical and immunologic phenotype.

In our registry, we identified mutations in 46 different genes (considering also DiGeorge syndrome micro deletions) and five previously undescribed genetic defects. The vast majority of the mutations were missense (*n* = 88, 57%) followed by 26 frameshift mutations, 16 splice-site defects, 10 non-sense mutations, and 14 large deletions. It is worth mentioning that large deletions mainly clustered in DOCK8 deficiency (*n* = 10), while the other types of mutations were more uniformly distributed among the different categories. The finding that missense mutations were the most common type resulting in PIDs was also documented in the Tunisian population (38%) while deletions accounted for almost 22% of all mutations (25).

Because the majority of PIDs are inherited as AR traits, the identification of patients with singular clinical and immunological phenotype within consanguineous families has been a crucial factor for the discovery of five novel disease-causing genes (*TRFC, DOCK2, MYSM1, NEIL3, RNF31*) in our registry. Families from areas with high rate of consanguineous marriages have been important for the identification of novel and complex phenotypes associated with PIDs (39). Importantly, the new genetic defects were discovered by using wide genome screening approaches like WES or WGS. These tools have already been shown to be effective diagnostic methods as first-line molecular assays patients suspected to have PIDs with no defect in known PID causing genes (40). Furthermore, these findings highlight the important role of NGS to unravel the genetic basis of novel PIDs. Our series shows that using sophisticated diagnostic methods can allow for substantial increase in the understanding of PID genetics, especially in the setting of inbred populations.

Studying patients with PIDs with parental consanguinity may not only lead to the discovery of novel disease-causing genes but also uncover novel inheritance patterns of known genes resulting in unexpected clinical phenotypes, or new phenotypes associated with known PID causing genes possibly caused by different mutations, different antigenic exposure and different genetic background. Despite the relatively large number of patients of our registry, we could not clearly identify novel mode of transmission or new clinical presentation for known genetic defects. However, it is worth mentioning that while AD heterozygous mutations are the most common way of inheritance of ALPS caused by *FAS* gene (41), two patients with ALPS in our cohort showed homozygous insertion in the same gene, inherited from consanguineous parents. This is consistent with what was recently reported cases from consanguineous populations (42).

Knowledge of the genetic defects responsible for many PIDs offers definite benefits in establishing a reliable diagnosis. Such information would also allow the establishment of a prognosis and the introduction of precision medical interventions. Some of the major barriers to widespread use of genetic testing for PIDs include relatively high costs, an often long turn-around time and difficulties in interpreting the results. To address these issues, efforts should be made to facilitate access to genetic testing (including WES), and to standardize and possibly simplify the language used in the genetic reports.

## Author Contributions

WA-H: development of the research concept and goals, design of methodology, data collection, writing the initial manuscript draft, approval of the submitted manuscript, and agreement to be accountable for the content of the work. MM, WB, and RC: performance the genetic testing, critical review of the manuscript, approval of the submitted manuscript, and agreement to be accountable for the content of the work. CK, YB, RG, and LN: supervision of genetic testing, critical review of the manuscript, approval of the submitted manuscript, and agreement to be accountable for the content of the work. JC and OMD: performance the genetic testing, contributed to the writing of the manuscript, critical review of the manuscript, approval of the submitted manuscript and agreement to be accountable for the content of the work.

### Conflict of Interest Statement

The authors declare that the research was conducted in the absence of any commercial or financial relationships that could be construed as a potential conflict of interest.
